# The Effect of Helicobacter Pylori Eradication on Liver Fat Content in Subjects With Non-Alcoholic Fatty Liver Disease: A Randomized Open-Label Clinical Trial

**DOI:** 10.5812/hepatmon.14679

**Published:** 2013-12-06

**Authors:** Raika Jamali, Alireza Mofid, Homayoon Vahedi, Rojin Farzaneh, Shahab Dowlatshahi

**Affiliations:** 1 Student Scientific Research Center, Tehran University of Medical Sciences, Tehran, IR Iran; 2 Internal Medicine Ward, Sina Hospital, Tehran University of Medical Sciences, Tehran, IR Iran; 3 Digestive Disease Research Institute, Tehran University of Medical Sciences, Tehran, IR Iran

**Keywords:** Non-alcoholic Fatty Liver Disease, Helicobacter Pylori, Insulin Resistance, Alanine Transaminase, Aspartate Aminotransferases

## Abstract

**Background:**

The role of Helicobacter pylori (HP) in the pathogenesis of nonalcoholic fatty liver disease (NAFLD) is unclear.

**Objectives:**

The aim of this study was to evaluate the effect of HP eradication on liver fat content (LFC), liver function tests (LFT), lipid profile, and homeostasis model assessment-IR (HOMA-IR) index in NAFLD.

**Patients and Methods:**

Dyspeptic patients with increased serum aminotransferase levels were enrolled in the study. The exclusion criteria were factors affecting serum aminotransferase or HP treatment strategy. Participants with persistent elevated serum aminotransferase level and ultrasound criteria for identification of fatty liver were presumed to have NAFLD. “NAFLD liver fat score” was used to classify NAFLD. Those with “NAFLD liver fat score” greater than -0.64 and positive results for urea breath test (UBT), were included. Lifestyle modification was provided to all participants. HP eradication was performed in intervention arm. LFC, fasting serum glucose (FSG), alanine aminotransferase (ALT), aspartate aminotransferase (AST), alkaline phosphatase (ALP), triglyceride (TG), cholesterol (CHOL), high and low-density lipoprotein (HDL, LDL), and HOMA-IR were checked at baseline and after that, at intervals of eight weeks and twenty four weeks.

**Results:**

One hundred (49 males) patients with the mean age of 43.46 (± 11.52) were studied. Repeated measure ANOVA showed a significant reduction in LFC, anthropometric measurements, and laboratory parameters (except for HDL) in the both groups during the study; however, no significant difference was observed between the groups.

**Conclusions:**

It seems that HP eradication per se might not affect LFC, LFT, lipid profile, and insulin resistance in dyspeptic NAFLD patients.

## 1. Background

The effect of gut microbiota on the development and progression of liver cell damage has recently come to interest. In this regard, helicobacter pylori (HP) deoxy-ribo-nucleic acid (DNA) was detected in patients with various etiologies of chronic liver disease (CLD) including nonalcoholic fatty liver disease (NAFLD) (1-4). Regardless of HP DNA existence in liver samples, no bacteria could be cultured (5). 

There are proposed mechanisms for the development of NAFLD in patients with HP infection. Taylor et al. expressed the earlier theory that HP species would produce a liver specific toxin that causes liver cell damage (6). Afterwards, it was developed that HP invasion to intestinal mucosa might increase gut permeability and facilitate the bacterial endotoxin passage via portal vein to the liver (7). Since HP treatment is not difficult and comparatively not expensive in most cases, discovering its role in diseases apart from the stomach could be of great importance for public health.

Despite accumulated evidence on the importance of HP on NAFLD pathogenesis , currently the way in which HP eradication would affect clinical as well as paraclinical state of patients with NAFLD is not well cleared (8-11).

## 2. Objectives

This study was designed to determine the short term effects of HP eradication on liver fat content, liver function tests (LFT), and metabolic profile (biochemical parameters and anthropometric measurements) in a sample of dyspeptic NAFLD patients. Considering that HP eradication in all infected patients is not recommended based on the current evidence, only dyspeptic patients were enrolled in the study.

## 3. Patients and Methods

### 3.1. Study Design

This study was designed as a randomized open-label clinical trial (ClinicalTrials.gov ID: NCT01876108). According to a predefined computer-generated block randomization table with a 1:1 allocation, each of the patients was assigned to treatment or control group. Participants filled out an informed written consent prior to enrollment. 

### 3.2. Subjects

Adult patients referred to the gastroenterology clinic of a general hospital for the evaluation of increased serum aminotransferase levels were selected. Serum alanine aminotransferase (ALT) and aspartate aminotransferase (AST) levels ≥ 40 units per liter (U/L) were considered elevated (12). Those patients with dyspepsia, positive results for anti HP antibody, and the evidence of fatty liver in ultrasonography were enrolled in the study (Step 1). The exclusion criteria were factors affecting serum aminotransferase or HP treatment strategy (Step 2) (13). All subjects with elevated serum aminotransferase levels in the first blood sampling were rechecked after two months (lead-in phase). Persistent elevated serum aminotransferase level was diagnosed based on either ALT or AST elevation in the second assessment. Participants with persistent elevated serum aminotransferase level and well-defined ultrasound criteria to identify fatty liver were presumed to have NAFLD (13). Considering the liver biopsy limitations, ‘NAFLD liver fat score’ was used in these enrolled patients to define NAFLD (14). Finally, those with NAFLD liver fat score greater than -0.64, and positive results for urea breath test (UBT) were included in the study (Step 3). 

### 3.3. Interventions

Lifestyle modification: lifestyle modification was the mainstay of treatment and was provided to all participants. It consisted of providing calorie-restricted diets and programmed physical activity to obtain ideal body weight. The protocol used for diet and exercise in our study was based on the “Guidelines for the diagnosis and management of nonalcoholic fatty liver disease: update 2010” (15). Rapid weight loss was avoided since it could deteriorate NAFLD (16). A dietitian who was blinded to the study protocol checked the participants, and controlled their daily calorie intake during the study. 

### 3.4. HP Eradication

The patients in treatment group received quadruple therapy with omeprazole (20 mg twice daily), amoxicillin (1 g twice daily), bismuth subcitrate (240 mg twice daily), and clarithromycin (500 mg twice daily) for two weeks (17). The participants were requested to take back their used packages and bottles of medications at the follow up visits every week. A gastroenterologist supervised this process to evaluate their compliance (by means of pill count) or any potential adverse effects.

### 3.5. Study Measurements

The percent of liver fat content was calculated based on the validated formula (14). A single radiologist blinded to patients’ clinical data, performed the liver ultrasonography. The echogenicity of the right lobe of liver was compared to the right kidney (in the sagittal view) with a 3.5 megahertz probe (Logiq 200 PRO, Tokyo, Japan) to identify fatty liver (18).

HP antibody (IgG) was measured using a quantitative enzyme-linked immunosorbent assay method (K5HPG, Radim Diagnostics, Rome, Italy). UBT was performed by Helioprobe test kit (Noster System, Stockholm, Sweden) to document HP infection and eradication. Eradication was investigated six weeks after the end of treatment (19). Waist circumference (WC) was measured at midpoint between the lower border of rib cage and the top of iliac crest at the end of expiration (20).

After a twelve-hour overnight fast, sera of the participants were tested to investigate fasting serum glucose (FSG), ALT, AST, alkaline phosphatase (ALP), triglyceride (TG), cholesterol (CHOL), low-density lipoprotein (LDL), and high-density lipoprotein (HDL) by enzymatic methods (13). Serum insulin concentration was measured based on a sandwich enzyme immunoassay using a commercially available kit (Biovendor, Brno, Czech Republic). Quantitative measurement of insulin resistance (IR) was performed using homeostasis model assessment-IR (HOMA-IR = fasting serum insulin × fasting serum glucose/22.5) (21). Diabetes mellitus (DM) was diagnosed according to either previous diagnosis of DM by a physician, or if FBS was equal or greater than 126 mg/dL, which was checked twice. The laboratory investigators were blinded to the case and control status of participants. 

### 3.6. Outcome Measures

Primary outcome measure was change from baseline liver fat content at 6 months. Secondary outcome measures were changes from baseline LFT, metabolic parameters, and anthropometric measurements at 6 months. 

### 3.7. Sample Size Calculation

A statistical power analysis was used to calculate the sample size. Considering the two-sided significant level (α) of 0.05 and the power of 90% (β = 0.1), a total sample size of 46 patients was determined to detect one percent inter-group difference in liver fat content. (For detailed information, please refer to http://hedwig.mgh.harvard.edu/sample_size/js/js_parallel_quant.html). The eradication rate for the regimen used for HP therapy was 95% in our earlier pilot trial (ClinicalTrials.gov ID: NCT01654549). To allow for a 5% possible dropout rate, 50 patients were recruited in each treatment group of the study. 

### 3.8. Statistical Analysis

Data was summarized as means ± SD for continuous, and number (percentage) for qualitative variables. Kolmogorov-Smirnov test was used to evaluate the normal distribution of the continuous variables. T test and Chi-square test were used to compare the mean values of continuous and categorical variable between the treatment groups. Repeated measure analysis of variance (ANOVA) was applied for comparing treatment groups regarding the changes of variables during the study. All statistical analyses were performed using SPSS version 17 (SPSS, Chicago, IL, The USA). The probability of the difference between the dependent and independent variables were considered significant if a two-tailed P value was less than 0.05. 

## 4. Results

### 4.1. Patients Enrolled

One hundred and twenty five patients suspected of having NAFLD were evaluated from July 2012 to July 2013. One hundred patients, with the mean age of 43.46 (± 11.52) years, were enrolled in the study. Table 1 represents patients’ characteristics. Reasons for leaving out were patient unwillingness to participate in the study (n = 12), normalization of ALT during the lead-in phase (n = 10), renal failure (n = 1), and pregnancy or lactation (n = 2). 

**Table 1.** The Baseline Characteristics of Participants

	Lifestyle modification plus *Helicobacter pylori* eradication	Lifestyle modification	P value
**Age, mean ± SD, y**	43.8 ± 11.3	43.1 ± 11.7	0.7
**Gender**			0.5
Male	22	27	
Female	26	23	
**Diabetes mellitus**			0.5
Present	22	22	
Absent	26	28	

### 4.2. HP Treatment Adverse Effects, Compliance and Eradication Rate 

Medications side effects necessitating dose reduction or discontinuation did not occur during the study. Pill counts during the follow up visits discovered a good adherence to therapy with the mean consumption rate of 94% of expected tablets (range from 90 to 98). Based on the UBT results, the eradication rate was 96%.

### 4.3. Laboratory and Anthropometric Findings

All the continuous variables were normally distributed at the baseline measurement. Table 2 shows the comparisons of LFC, anthropometric measurements, and laboratory parameters according to the treatment groups during the study. There was no significant difference regarding the studied variables between the treatment groups at baseline, eight weeks, and twenty-four weeks. 

**Table 2.** The Comparison of Liver Fat Content, Anthropometric Measurements, and Laboratory Parameters Between the Treatment Groups During the Study ^a^

	Lifestyle modification plus *H. pylori* eradication	Lifestyle modification	P value
**Liver fat content, mean ± SD**			
Baseline	12.5 ± 7.2	12.2 ± 7.9	0.8
Eight weeks	10.7 ± 6.1	10.2 ± 6.9	0.7
Twenty four weeks	8.9 ± 5.1	8.6 ± 6.1	0.7
**Waist Circumference, mean ± SD, cm**			
Baseline	101.2 ± 2.8	100.7 ± 2.6	0.3
Eight weeks	101.1 ± 2.7	100. 6 ± 2.5	0.3
Twenty four weeks	98.1 ± 1.7	98.2 ± 1.7	0.8
**Weight, kg**			
Baseline	87.6 ± 12.1	88.5 ± 8.5	0.6
Eight weeks	86.8 ± 11.5	87.6 ± 8.7	0.7
Twenty four weeks	79.4 ± 9.3	80.1 ± 6.8	0.6
**Body mass index, kg/m** ^**2**^			
Baseline	31.2 ± 4.1	30.4 ± 4.2	0.3
Eight weeks	30.9± 3.9	30.1 ± 4.1	0.2
Twenty four weeks	28.3 ± 3.2	27.5 ± 3.5	0.2
**Aspartate aminotransferase, U/L**			
Baseline	46.5 ± 19.9	46.4 ± 21.2	0.9
Eight weeks	38.3 ± 15.2	39.2 ± 18.7	0.8
Twenty four weeks	32.4 ± 13.0	33.4 ± 15.4	0.7
**Alanine aminotransferase, U/L**			
Baseline	68.6 ± 26.1	73.5 ± 30.8	0.4
Eight weeks	53.7 ± 30.5	53.3 ± 33.3	0.9
Twenty four weeks	41.9 ± 22.1	41.3 ± 25.6	0.9
**Alkaline phosphatase, U/L**			
Baseline	182.3± 58.5	191.8 ± 50.9	0.3
Eight weeks	172.8 ± 51.6	187.4 ± 48.5	0.1
Twenty four weeks	162.7 ± 41	176.5 ± 41.4	0.1
**Triglyceride, mg/d**L			
Baseline	174.8 ± 103.2	155.8 ± 83.4	0.3
Eight weeks	137.5 ± 79.0	124.4 ± 52.2	0.3
Twenty four weeks	119.9 ± 48.1	115.8 ± 40.3	0.6
**Cholesterol, mg/d**L			
Baseline	180.8 ± 31.9	183.3 ± 40.3	0.7
Eight weeks	171.5 ±26.9	173.3 ± 31.7	0.7
Twenty four weeks	167.2 ± 20.8	168.0 ± 23.1	0.8
**Low density lipoprotein, mg/d**L			
Baseline	101.8 ± 25.7	108.7 ± 37.1	0.2
Eight weeks	97.1 ± 23.3	102.4 ± 29.7	0.3
Twenty four weeks	91.5 ± 19.3	93.8 ± 21.0	0.5
**High density lipoprotein, mg/d**L			
Baseline	44.5 ± 5.7	43.1 ± 5.8	0.2
Eight weeks	46.9 ± 5.5	46.0 ± 7.1	0.5
Twenty four weeks	51.7 ± 7.0	51.0 ± 8.1	0.6
**Fasting plasma glucose, mmol/L**			
Baseline	5.9 ± 0.8	5.8 ± 0.8	0.5
Eight weeks	5.5 ± 0.5	5.5 ± 0.6	0.6
Twenty four weeks	5.2 ± 0.7	5.3 ± 0.6	0.5
**HOMA-IR ** ^**b**^			
Baseline	5.2 ± 1.9	5.0 ± 2.1	0.6
Eight weeks	4.3 ± 1.5	4.1 ± 1.7	0.6
Twenty four weeks	3.6 ± 1.4	3.5 ± 1.6	0.7

^b^ Abbreviation: HOMA-IR, Homeostasis model assessment-insulin resistance

^a^Data presented as mean ± standard deviation

Table 3 provides results of repeated measure ANOVA with Greenhouse-Geisser correction. This analysis determined that mean studied parameters differed statistically significantly between the time points. Post hoc tests using the Bonferroni correction revealed that all treatment groups elicited statistically significant reduction in LFC, anthropometric measurements, and laboratory values (except for HDL) from baseline to the end of study. 

**Table 3.** The Comparison of Liver Fat Content, Anthropometric Measurements and Laboratory Parameters Change Regarding the Treatment Groups During the Study ^a^

	Pair wise comparison between baseline and the end of treatment	
	**Mean **±** SD**	**P value**
**Liver fat content, %**	3.5 ± 0.3	< 0.01
**Waist circumference, cm**	2.7 ± 0.2	< 0.01
**Weight, kg**	8.2 ± 0.3	< 0.01
**Body mass index, kg/m** ^**2**^	2.9 ± 0.1	< 0.01
**Aspartate aminotransferase, U/L**	13.5 ± 1.1	< 0.01
**Alanine aminotransferase, (U/L)**	29.4 ± 2.0	< 0.01
**Alkaline phosphatase, U/L**	17.4 ± 2.3	< 0.01
**Triglyceride, mg/d**L	47.4 ± 6.2	< 0.01
**Cholesterol, mg/d**L	14.4 ± 2.1	< 0.01
**Low density lipoprotein, mg/d**L	12.5 ± 1.6	< 0.01
**High density lipoprotein, mg/d**L	-7.4 ± 0.6	< 0.01
**Fasting plasma glucose, mmol/L**	0.6 ± 0.0	< 0.01
**Fasting serum insulin, mU/L**	3.6 ± 0.3	< 0.01
**HOMA-IR ** ^**b**^	1.4 ± 0.1	< 0.01

^b^ Abbreviation: HOMA-IR, Homeostasis model assessment-insulin resistance

^a^Negative values represent the increase of the parameter at that interval

No statistically significant difference was observed in the changes of mean LFC, laboratory values, and anthropometric measurements between the treatment groups at the study interval (All P values > 0.05 in between-subject effects model)

## 5. Discussion

To the best of our knowledge, this is the first randomized open-label clinical trial which evaluated the effect of HP eradication on LFC, LFT, and metabolic parameters in NAFLD patients. According to the result of this study, HP eradication per se might not affect LFC, LFT, lipid profile, insulin resistance, and anthropometric measurements in dyspeptic NAFLD patients.

Liver ultrasonography has some limitations for diagnosis and grading the severity of NAFLD (18). However, its availability and cost effectiveness makes it an appropriate tool for fatty liver screening. We used this method to evaluate fatty liver in those with persistent elevated liver enzyme level. Performing the procedure by a single radiologist avoided inter-observer variability. Considering the possible complications and poor patient acceptance, we used “NAFLD liver fat score” and did not perform liver biopsy to document NAFLD diagnosis. Sensitivity and specificity of the values greater than -0.64 are 86% and 71% respectively, to predict NAFLD (14). 

To increase the feasibility and reproducibility of the study, we applied a valid formula to measure liver fat content. A previous study showed the validity of this equation to predict liver fat content considering proton magnetic resonance spectroscopy (PMRS) as the gold standard (14). There was a significant high correlation between liver fat content identified by PMRS and liver fat content calculated by the above formula (14) (r = 0.7, P < 0.0001).

Recently the role of HP in extragastric diseases has come to interest. There is a large body of evidence showing the association of HP and CLD (22-26). The prevalence of HP like DNA in the liver tissue samples of patients with CLD was significantly higher than the patients with metastatic adenocarcinoma (2). In the study of Ponzetto et al. the prevalence of HP antibody (IgG) was higher in males with HCV associated cirrhosis compared to age-matched male blood donors (23). The genomic sequences corresponding to HP were determined in the liver tissues of patients with HCC and cirrhosis. It was proposed that HP might be implicated in the progression of cirrhosis in patients with HCV infection (24). 

Specific studies regarding HP and NAFLD are increasing. The presence of HP DNA in one sample of liver tissue from a NAFLD patient was a novel finding in 2008 (3). Aller et al. showed that probiotics might decrease markers of lipid peroxidation and improve liver function tests (LFT) in NAFLD (27). Solga et al. reported that probiotics protect gut epithelial cells from the adhesion and invasion of HP (28). Therefore, it can be concluded that probiotics induce their therapeutic effects in NAFLD via avoidance of intestinal mucosa destruction by HP. It was suggested that HP infection was one of the risk factors for the development of NAFLD (25). The study by Polyzos et al. compared biopsy proven NAFLD patients with matched healthy controls (26). The anti HP antibody (IgG) was higher in NAFLD than controls. This study recommended that HP infection eradication might have therapeutic perspectives in NAFLD treatment. 

Lifestyle modification to control daily calorie intake is already considered as the gold standard care in NAFLD patients. We applied this strategy to all participants. Obtaining the ideal body weight resulted in the sustained improvement of biochemical (serum liver enzymes and insulin level) and histological findings in NAFLD (29). This is in agreement with the results of our study that showed the significant reduction of LFC, LFT, and metabolic indices (FSG, lipid profile, HOMA-IR, and anthropometric measurements) in both treatment groups during the study.

In our study, changes in LFC and LFT were not significantly different in HP eradicated group from controls who did not receive eradication treatment. This finding is somehow in accordance with the result of Stalke et al. (1). They reported no correlation between LFT and identification of HP DNA in the liver biopsy samples of CLD patients. 

We showed that HP eradication had no additional effect on the metabolic indices changes. It was suggested that gut microbiota might regulate IR (30). Currently, there are controversial results about the effect of HP on insulin resistance. The positive correlation of HP with the metabolic syndrome and the inverse correlation with morbid obesity were reported (8-11). Gunji et al. studied a large sample of Japanese general population (8). HP seropositivity was higher in those with metabolic syndrome than the controls. They suggested that HP infection could be associated with metabolic syndrome. On the other hand, there are reports showing the enhanced risk of obesity following HP cure (10, 11). These studies proposed that increased Ghrelin following HP treatment improves the appetite and leads to weight gain. We believe that the controversial results of the above studies could be due to the difference in the duration of HP infection, and the type of gastritis in the studied populations. 

The results of this study are compatible with our previous pilot trials on the effect of HP eradication in nondiabetic and diabetic patients with NAFLD. (ClinicalTrials.gov ID: NCT01654549 and NCT01712711 respectively).

### 5.1. The limitations of Study and Recommendation for the Future Studies

Since there is not enough evidence to show the benefit of HP eradication in all infected patients, only dyspeptic NAFLD patients were included in this study. Therefore, the results cannot be generalized to all NAFLD patients. Another limitation of this study was the lack of performing liver biopsy to define the histologic response. Comparing the effect of HP eradication on biochemical and histological changes in NAFLD patients (including non-dyspeptic) with longer follow up duration is recommended.

### 5.2. Summary

This randomized open-label clinical trial was performed on one hundred dyspeptic NAFLD patients who were randomly assigned to lifestyle modification alone or lifestyle modification plus HP eradication treatment. A dietitian checked the daily calorie intake. HP eradication treatment was performed by standard quadruple therapy for 2 weeks. HP eradication rate was 96% in this sample of NAFLD patients. A comparison was performed between successful eradication group, and those who did not receive HP eradication. Repeated measure ANOVA showed a significant reduction in LFC, anthropometric measurements, and laboratory parameters (except for HDL) in the both groups during the study; however, no significant difference was observed between the two groups.

Within the limitations of this study, it can be concluded that HP eradication in dyspeptic NAFLD patients did not provide any additional improvement in LFC, LFT, FSG, lipid profile, IR, and anthropometric measurements compared to lifestyle modification alone.
